# Evaluation of Knowledge, Attitudes and Practices for Hepatitis B Virus Infection Among Primary Healthcare Physicians in Georgia

**DOI:** 10.1111/jvh.14011

**Published:** 2024-10-10

**Authors:** Mamuka Zakalashvili, Sophia Surguladze, Davit Baliashvili, Jaba Zarkua, Tata Avalishvili, Elene Tsirdava, Mariam Tsodolishvili, David Metreveli, Natia Shavgulidze, Irina Tskhomelidze, Shaun Shadaker, Maia Tsereteli, Paige A. Armstrong, Senad Handanagic

**Affiliations:** 1 Medical Center Mrcheveli, Tbilisi, Georgia; 2 The Task Force for Global Health, Tbilisi, Georgia; 3 Central Republican Hospital, Tbilisi, Georgia; 4 Centers for Disease Control and Prevention, Atlanta, Georgia, USA; 5 National Center for Disease Control and Public Health Georgia, Tbilisi, Georgia

**Keywords:** Georgia, hepatitis B, KAP, primary healthcare workers, viral hepatitis

## Abstract

A nationwide serosurvey among adults in 2021 showed a 2.7% (95% confidence interval [CI]: 2.3%–3.4%) prevalence of hepatitis B. Our analysis evaluates knowledge, attitudes and practices (KAP) for hepatitis B virus (HBV) infection among primary healthcare physicians (PHPs) in Georgia. We randomly selected 550 PHPs from medical facilities in Georgia's six largest cities. Using bivariate ordinal regression, we assessed the association of socio-demographic factors with an ordinal knowledge score (low/middle/high). Multivariable logistic regression was performed to calculate adjusted odds ratios (aOR) and 95% CI to determine associations between HBV knowledge score and practices. Of 550 selected PHPs, 506 (92.0%) agreed to participate. Among them, 62.8% scored in the medium or high knowledge tertiles, 72.7% were confident in diagnosing HBV infection, 37.3% were confident in managing patients with hepatitis B; 47.4% reported being screened for and 26.2% reported being vaccinated against HBV infection. Compared to those with low knowledge scores, PHPs with a high score were less likely to recommend activities not supported by evidence, such as: the use of ‘hepatoprotective’ medications (aOR 0.43, 95% CI 0.25–0.73), caesarean sections (aOR 0.47, 95% CI 0.27–0.82) and withholding breastfeeding (aOR 0.57, 95% CI 0.34–0.96) to prevent HBV transmission. The majority of PHPs were confident in diagnosing HBV infection, but only one in three were confident in managing patients with hepatitis B. PHPs with higher HBV knowledge were less likely to provide inaccurate instructions to their patients. These findings will help to develop awareness and education campaigns supporting HBV elimination in Georgia.

## 1 ∣ Introduction

Viral hepatitis is one of the leading causes of death, with 296 million people worldwide having hepatitis B, according to World Health Organization (WHO) estimates in 2019 [1]. In 2016, WHO member states adopted the Global Health Sector Strategy for viral hepatitis elimination. The goal is to reduce the incidence of chronic infections by 90% (95% for hepatitis B and 80% for hepatitis C) and mortality by 65% (compared to 2015 levels), eliminating hepatitis B and hepatitis C as public health threats [2]. Despite some improvements in the diagnosis and treatment of hepatitis B and hepatitis C, progress towards these global elimination goals is suboptimal, with hepatitis B testing and treatment rates, in particular, lagging behind [1].

In 2015, the country of Georgia initiated a national hepatitis C virus (HCV) elimination programme, which also addressed hepatitis B virus (HBV) infections in specific populations. Nevertheless, hepatitis B remains a significant burden in Georgia. Two nationwide serosurveys estimated an HBV surface antigen (HBsAg) prevalence of 2.9% and 2.7% in 2015 and 2021, respectively, showing a stable burden of chronic HBV infection among adults [3-5]. Georgia has already achieved some HBV elimination targets, including reducing mother-to-child transmission (MTCT) through universal hepatitis B birth dose administration introduced in 2003 with consistent coverage of > 90% since 2012 and high rates of childhood vaccination coverage [3, 5]. The 2021 national serosurvey demonstrated a low prevalence of HBsAg (0.03%) among children aged 5–17 years and showed that Georgia has reached the European region hepatitis B control target and the WHO impact target for elimination of MTCT [5]. Screening of blood and blood products for hepatitis B has been implemented in all blood banks since 1997 [6].

Other hepatitis B control efforts in Georgia are limited, aside from hepatitis B vaccination in children and testing of blood donations. Furthermore, there are gaps in diagnosing and managing persons with chronic HBV infection, including limited awareness about HBV infection among the general population and dental workers and financial barriers to accessing HBV diagnostics, management, and surveillance [3, 7, 8]. The HBV care continuum for the most part is privatised; generally, patients are independently seeking care for HBV in specialised clinics.

To initiate efforts towards HBV elimination and management in the country, there is a need to evaluate current knowledge, attitudes and practices (KAP) regarding HBV infection among primary healthcare physicians (PHP). Chronic HBV infection is usually asymptomatic and proper recommendations and patient education are necessary to reduce morbidity and mortality [9]. Given that PHPs are the most readily accessible healthcare provider for most people in Georgia, their role becomes especially important in the context of HBV elimination.

This study aimed to evaluate and identify gaps in the KAP related to HBV infection among Georgian PHPs. These findings will help guide the development of education and training campaigns and can be taken into consideration when developing a national programme and action plan for hepatitis B elimination in Georgia.

## 2 ∣ Materials and Methods

### 2.1 ∣ Study Population

A sample size calculation was performed to estimate the frequencies in the PHP population. The population size of PHPs, which includes licensed physicians in Internal Medicine or Family Medicine, was estimated to be 2500. This is the first study exploring the KAP related to HBV infection in Georgia, so in calculating sample size, we used the prevalence of main outcomes (e.g., percentage of PHP who recommend screening for HBV infection to their patients) of 50% to maximise the sample size. The precision level was equal to 5% and the confidence interval was set to 95%. With a design effect of 1.5, the minimum number of subjects who should participate in the survey for the above-defined characteristics was 500. Considering the expected non-response rate of no more than 10%, we attempted to enrol 550 survey participants. The inclusion criteria were active, certified PHPs who had ongoing practice from a national or private hospital or clinic and were not specialised in HBV care.

### 2.2 ∣ Selection of Primary Healthcare Facilities

The study's clusters (the primary sampling units) were represented by six cities in the country (Tbilisi, Telavi, Rustavi, Kutaisi, Zugdidi and Batumi), covering the 6 largest regions out of the total 11 regions in Georgia. The secondary sampling units were the hospitals/clinics within the designated cities, which were selected from the list of medical facilities in the chosen regions by probability proportional to size.

The sampling frame of the medical facilities was the total number of PHPs working in each facility. The total number of selected medical facilities was 50 and 11 PHPs were selected from each facility. The selection of PHPs within the identified hospitals/clinics was done by randomly sampling from the lists of PHPs obtained from the hospital/clinic's authorities. The PHPs were invited for the interview at their workplace and enrolment continued until the target number of participants was reached at a given clinic.

### 2.3 ∣ KAP Questionnaire and Administration

The study field team administered a paper form survey to assess KAP related to HBV infection (see Appendix S1).

Knowledge regarding HBV infection was assessed from 28 questions split into four categories: (1) general knowledge; (2) knowledge of risk factors; (3) knowledge of diagnosis and treatment; and (4) knowledge of vaccination. Attitudes and perceptions comprised 14 questions that focused on physicians' confidence in diagnosing and managing HBV infection, perceptions about treatment for and vaccination against hepatitis B and stigma associated with HBV infection. There were 18 practice-related questions in the survey, which focused on hepatitis B screening and vaccination, recommendations and counselling provided by the physicians to patients, maternal and child care and pre-scription of off-label medications for HBV infection. The latter medications refer to drugs known as ‘hepatoprotective medications’ that usually refer to off-label, unapproved drugs for the management of patients with hepatitis B, such as some herbal remedies (silymarin, Cynara scolymus, etc.) or antioxidants (ademetionine, L-carnitine, L-ornithine, glutathione, lecithin, etc.) [10, 11]. While these medications have not been documented to have a negative effect on the liver, their benefits have also not been well established [10-13].

**FIGURE 1 ∣:**
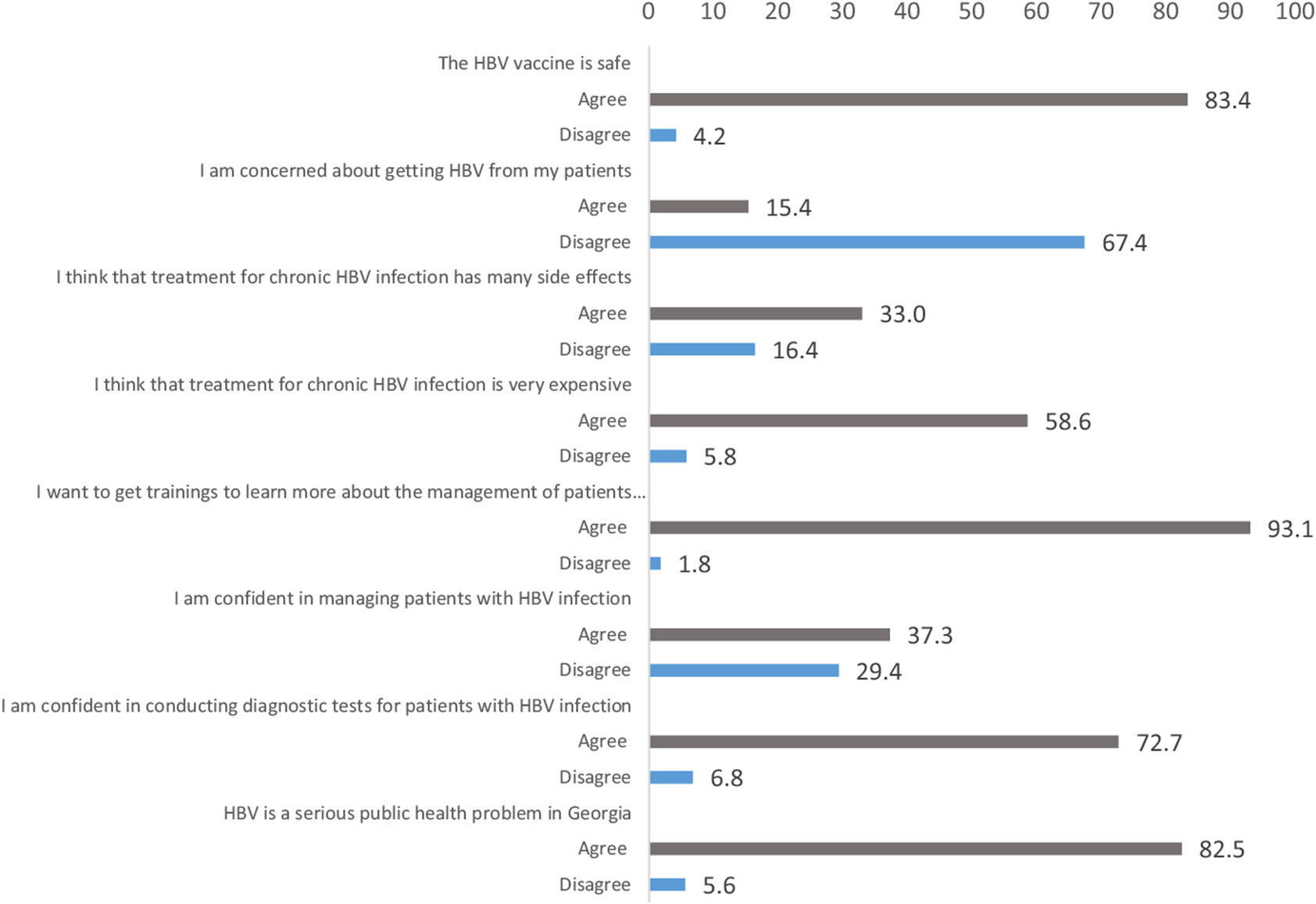
Primary healthcare physicians' attitudes and perception of hepatitis B, Georgia, 2022. Possible answers for each question were as follows: strongly agree, agree, neutral, disagree, strongly disagree and do not know. For this figure, we merged answers agree and strongly agree into agree and disagree and strongly disagree into disagree. Values do not add to 100% because we omitted do not know and neutral responses. Complete responses are presented in Table S1.

Questions asking about attitudes and perception of hepatitis B had six potential answers: strongly agree; agree; neutral; disagree; strongly disagree; and do not know (Table S1). To summarise the findings and present the results in Figure 1, we merged answers agree and strongly agree into agree and disagree and strongly disagree into disagree and we omitted the answers ‘neutral’ and ‘don't know’ from Figure 1.

### 2.4 ∣ Data Analysis

Statistical analysis for this study was conducted using the R statistical software with statistical significance being determined at an alpha level of 0.05. The knowledge level of each participant was assessed by calculating individual knowledge scores by counting each correct answer as one point; total knowledge scores ranging from 0 to 28 were calculated (based on the number of correct answers in the ‘knowledge’ section of the survey). The knowledge scores of the participants were then classified as low (0–16), middle (17–19) and high (20–28) based on the tertile distribution.

Bivariate ordinal regression was used to calculate odds ratios (OR) with 95% confidence intervals (CI) to assess the association of socio-demographic factors with the ordinal knowledge score (low, middle and high). Additionally, multivariable logistic regression was used to assess the associations between hepatitis B knowledge score and practices while controlling for age and sex.

### 2.5 ∣ Ethics

The study was approved by the NCDC's institutional review board and participants gave informed consent with a unique code linked to their questionnaire; no names were recorded.

## 3 ∣ Results

Overall, 506 PHPs agreed to participate in the study, with a response rate of 92.0% (506/550). Of these, 93.1% were female, 63.8% were 50 years or older and 34.7% lived in the capital city of Tbilisi (Table 1). The majority of PHPs (72.6%) had more than 15 years of work experience, 76.3% practiced family medicine and 75.1% saw 10 or fewer patients with HBV infection annually.

### 3.1 ∣ HBV-Related Knowledge

Of 506 interviewed PHPs, 318 (62.8%) scored in the medium or high tertile, corresponding to a median of 18 (standard deviation [SD] = 3.85) out of 28 correct answers.

As shown in Table 2, 69.6% of participants correctly identified that hepatitis can be caused by viruses, alcohol or drugs and 31.0% correctly listed all potential complications of chronic HBV infection, while 62.6% incorrectly responded that cirrhosis and cancer were the only consequences of HBV infection. Only one participant (0.2%) precisely indicated all populations at risk of HBV infection, while 90.5% identified only persons on haemodialysis as a population at risk for HBV infection. Only 39.5% of participants correctly identified hepatitis B surface antigen (HBsAg) as the primary diagnostic test for chronic HBV infection.

For HBV transmission-related questions, sexual intercourse, sharing needles or syringes and MTCT were correctly identified by 54.4%, 74.0% and 42.8% of participants, respectively; 17.0% of participants correctly identified all modes of transmission. Symptoms of acute HBV infection were correctly identified by 81.2% of PHPs. Forty-three per cent (43.4%) accurately identified that there are no contraindications for hepatitis B vaccination. For the prevention of MTCT, 49.3% of participants correctly discerned that it is recommended to provide hepatitis B immune globulin and hepatitis B birth dose vaccine and that caesarean sections and withholding of breastfeeding are not needed.

### 3.2 ∣ HBV-Related Attitudes and Perceptions

A large majority (82.5%) of PHPs agreed that HBV infection is a serious public health threat in Georgia. As shown in Figure 1, 72.7% of participants claimed they were confident in conducting diagnostic tests for HBV infection and 37.3% were confident in managing patients with hepatitis B. The vast majority (93.1%) expressed a desire to engage in training to improve their knowledge of HBV. More than half (58.6%) of participants believed that HBV treatment was very expensive and 33.0% believed that it had many side effects. There was a concern about contracting HBV infection from patients for 15.4% of PHPs and 83.4% of participants agreed that the hepatitis B vaccine was safe.

### 3.3 ∣ HBV-Related Practices

Regarding practices pertaining to HBV infection, 47.4% of participants reported that they were screened for hepatitis B and 26.2% reported being vaccinated against HBV infection (Table 3). When asked about recommendations given to their patients with HBV infection, the following percentage provided inaccurate advice: 37.2% of participants recommend avoiding sharing food and utensils, 28.2% recommend restricting physical activity, 40.7% recommend taking hepatoprotective medications and 47.2% recommend a caesarean section to pregnant patients with HBV infection. For screening, 92.0% of participants recommended HBV screening to their patients, 86.4% recommended screening and vaccination for sexual partners of patients with HBV infection and 84.3% recommended screening family members and close contacts of patients with hepatitis B.

### 3.4 ∣ Determinants of Adequate Hepatitis B Knowledge and Practices

Participant's sex and number of years working as a physician were not significantly associated with the total overall knowledge score (Table 4). Participants with high knowledge scores were less likely to recommend a caesarean section for HBV-positive pregnant women than those who received a low score (aOR 0.47, 95% CI 0.27–0.82) (Table 5). PHPs who received a middle score or a high score were less likely to wrongly pre-scribe hepatoprotective medications to their patients with HBV infection (aOR 0.55, 95% CI 0.31–0.96 and aOR 0.43, 95% CI 0.25–0.73, respectively) compared to those with a low score. Those with high knowledge scores were less likely to recommend avoiding breastfeeding to mothers with HBV infection (aOR 0.57, 95% CI 0.34–0.96) than those who received lower knowledge scores. Receiving a middle or high knowledge score was not significantly associated with being vaccinated against hepatitis B (aOR 1.55, 95% CI 0.92–2.61 and aOR 1.25, 95% CI 0.73–2.14 respectively) compared to those with low scores.

## 4 ∣ Discussion and Conclusion

Our study found that most PHPs had either a medium or high level of knowledge of hepatitis B and PHPs with higher knowledge scores were more likely to provide accurate recommendations to their patients. We did not find a significant association between knowledge scores and self-reported vaccination for hepatitis B. However, significant gaps were identified in the knowledge of prevention, diagnostics and management of HBV infection.

The results from the attitude questionnaires were unexpected. Many participants considered themselves capable of ordering diagnostic testing for HBV infection for patients with HBV infection, though less than half of them knew that HBsAg is the primary diagnostic test for chronic HBV infection; very few PHPs were confident in the management of HBV infection (follow-up of patients and administration of anti-viral treatment). We also found that approximately 40% of PHPs recommend ‘hepatoprotective’ medications, which are widely available in Georgia but are off-label and not approved for the management of HBV infection.

Aside from our study, based on available data, current practices and expert opinion, it is accepted that there is a lack of knowledge about HBV among the general population, patients at higher risk and non-HBV-specialised physicians in Georgia [5, 14]. A study conducted in 2010 on the barriers to hepatitis B vaccination coverage among healthcare workers in Georgia showed that only 12% of healthcare workers were vaccinated, which is the lowest according to the available data in the literature on healthcare worker vaccination overall [14]. Low vaccination coverage was also observed in our study, with only one in four of the participants reported being vaccinated against hepatitis B. We also found that the level of knowledge about hepatitis B was not associated with a higher likelihood of being vaccinated.

Similar to our findings, studies among healthcare workers in Saudi Arabia, Uganda, Pakistan, several African countries and Mongolia showed they lack knowledge in specific areas such as modes of transmission and prevention and post-exposure management [15-21]. This highlights the need for improving the training of healthcare workers and educating medical students on hepatitis B. In Vietnam, healthcare workers who received training on hepatitis B 2 years before a KAP survey had significantly better knowledge scores on hepatitis B compared to those who did not receive recent training [22].

Given the benefits of training healthcare workers demonstrated in other countries and that over 90% of PHPs in our study were amenable to training, building the capacity of PHPs in Georgia to provide care for patients with hepatitis B should be considered. Increasing PHPs awareness of the guidelines and risks concerning using hepatoprotective medications is necessary for effective hepatitis B care. Focus should also be placed on increasing vaccination coverage among PHPs and given that the level of knowledge was not associated with vaccination, efforts should also be made to identify the factors contributing to such low vaccination coverage.

## 5 ∣ Strengths and Limitations

Stratified sampling and probability proportional to size sampling were used in the current study to maximise generalisability to the PHP population in Georgia. The selection of six cities covering the 6 largest of the total 11 regions of Georgia and many of the PHPs working at these hospitals also reside in the rural areas of those regions; this ensures that the sample is diverse and captures the variations in KAP across the country—both in rural and urban areas. The strength of this study is the high response rate (92%), which could be partially due to the anonymous nature of the survey. Nevertheless, this study also has limitations. Our study sample was 93% female, which may not be representative of the sex distribution of PHPs in Georgia. However, anecdotal evidence suggests that a large majority of PHPs are female, but we do not have data to confirm this. Similar studies in Georgia have found a higher percentage of female physicians participating in KAP surveys (e.g., 84% in the KAP survey among gynaecologists) [23]. Given the large pre-dominance of female PHPs in our sample, the representativeness of results to all PHCs needs to be done with caution. Furthermore, recall bias and social desirability bias might have affected the accuracy of the self-reported data collected from the study participants.

In conclusion, this study found that PHPs' knowledge in some areas was high (such as regarding the symptoms of acute HBV infection). Still, gaps were identified in awareness of HBV transmission routes and effective prevention interventions. Many PHPs were willing to receive further training to improve their knowledge about hepatitis B. Nevertheless, there were gaps in their practices, such as low vaccination rates among PHPs and a high percentage recommending unnecessary interventions like caesarean sections for the prevention of MTCT of HBV. These findings should be followed by comprehensive educational and training campaigns targeting improvement in patient care, reducing the HBV transmission rate and following WHO viral hepatitis elimination recommendations to advance the elimination of viral hepatitis in Georgia.

## Supplementary Material

Supplementary table

Appendix

## Figures and Tables

**TABLE 1 ∣:** Demographic characteristics of selected primary healthcare physicians, Georgia 2022.

Characteristics	Primary health care physicians (*N* = 506)	
	*n*	%
Sex		
Male	35	6.9
Female	469	93.1
Missing	2	
Age, years		
< 30	10	2.0
30–39	46	9.1
40–49	127	25.1
50–59	153	30.2
≥ 60	170	33.6
Region of residence		
Tbilisi	175	34.7
Batumi	87	17.2
Kutaisi	72	14.3
Zugdidi	53	10.5
Rustavi	50	9.9
Telavi	36	7.1
Other	32	6.3
Missing	1	
Years of working as a physician		
≤ 5	25	5.0
6–10	54	10.8
11–15	47	9.4
> 15	364	72.6
Refused to answer	11	2.2
Missing	5	
Number of patients with hepatitis B seen annually		
0–10	380	75.1
11–50	89	17.6
51–100	9	1.8
> 100	28	5.5
Specialty		
Family medicine	386	76.3
Internal medicine	81	16.0
Infectious disease	4	0.8
Gastroenterology	1	0.2
Other	34	6.7

Abbreviation: HBV, hepatitis B virus.

**TABLE 2 ∣:** Primary healthcare physicians' knowledge of hepatitis B, Georgia 2023.

Questions	Primary health care physicians (*N* = 506)	
	*n*	%
Which of the below can cause hepatitis (inflammation of the liver)?		
Virus	140	27.8
Alcohol	5	1.0
Drugs	1	0.2
All above mentioned^a^	350	69.6
None of the above	4	0.8
Do not know	2	0.4
Refuse to answer	1	0.2
Missing	3	
Chronic HBV infection can cause		
Liver cirrhosis and cancer	317	62.6
Arthritis	2	0.4
Vasculitis	2	0.4
Renal failure	1	0.2
All mentioned^a^	157	31.0
None of the above	6	1.2
Other	4	0.8
I do not know	17	3.4
Populations at risk of HBV infection are		
Healthcare workers	4	0.8
People with multiple blood transfusions	16	3.2
People who inject drugs	17	3.3
Commercial sex workers	2	0.4
Children born to HBV-positive mothers	0	0.0
Persons on haemodialysis	456	90.5
All above mentioned^a^	1	0.2
None above mentioned	3	0.6
I do not know	5	1.0
Missing	2	
HBV can be transmitted via^b^		
Droplets		
No^a^	487	97.8
Food		
No^a^	486	97.2
Blood		
Yes^a^	476	95.2
Sexual contact		
Yes^a^	272	54.4
Handshake with an infected person		
No^a^	495	99.0
Sharing household objects like razors or toothbrushes		
Yes^a^	180	36.0
Sharing needles or syringes		
Yes^a^	370	74.0
Touching items in public places		
No^a^	495	99.0
Mother to child during birth		
Yes^a^	214	42.8
Identified all modes of transmission correctly	85	17.0
What test is usually used first to confirm current HBV infection?		
Anti-HBc (hepatitis B core antibodies)	94	18.6
HBsAg (hepatitis B surface antigen)^a^	200	39.5
HBeAg (hepatitis B envelope antigen)	10	2.0
Anti-HBs (hepatitis B surface antibody)	72	14.2
PCR HBV-DNA (PCR for identification of viral DNA)	35	6.9
I do not know	55	10.9
Refused to answer	40	7.9
What can prevent MTCT of HBV?		
Caesarean section	65	12.9
Hepatitis B immunoglobulin within the first 24 h after birth and timely birth dose vaccination^a^	249	49.3
Withholding breastfeeding	3	0.6
All above mentioned	96	19.0
None above mentioned	21	4.2
Do not know	29	5.7
Refused to answer	42	8.3
Missing	1	
Which of the following are contraindications to HBV vaccination?		
Allergic rhino-sinusitis	2	0.4
Neurologic disorders	8	1.6
Liver diseases	89	17.6
Age > 60	14	2.8
All above mentioned	58	11.5
None above mentioned^a^	219	43.4
Do not know	57	11.3
Refused to answer	58	11.4
Missing	1	
Acute HBV infection can be presented with		
Jaundice	34	6.7
Nonspecific symptoms (fever, nausea, fatigue, etc)	31	6.1
Fulminant hepatitis (ascites, encephalopathy, coagulopathy, etc.)	0	0.0
Without symptoms	20	4.0
All above mentioned^a^	411	81.2
I do not know	5	1.0
Refused to answer	5	1.0

Abbreviation: HBV, hepatitis B virus.

a Correct answer.

b Only correct answers presented.

**TABLE 3 ∣:** Primary healthcare physicians' HBV-related practices, Georgia 2022.

Questions	Primary health care physicians (*N* = 506)	
	*n*	%
Have you been screened for hepatitis B?		
Yes	239	47.4
No	218	43.3
Not sure	15	3.0
Refused to answer	32	6.3
Missing	2	
Have you been vaccinated against hepatitis B?		
Yes	132	26.2
No	314	62.4
Not sure	25	5.0
Refused to answer	32	6.4
Missing	3	
Do you recommend patients with HBV infection to avoid sharing food/utensils/water with others?		
Yes	187	37.2
No	232	46.1
Not sure	56	11.1
Refused to answer	28	5.6
Missing	3	
Do you recommend restricted physical activity to patients with HBV infection?		
Yes	142	28.2
No	249	49.5
Not sure	92	18.3
Refused to answer	20	4.0
Missing	3	
Do you recommend caesarean section for HBV-positive pregnant women?		
Yes	238	47.2
No	121	24.0
Not sure	120	23.8
Refused to answer	25	5.0
Missing	2	
Do you recommend to your patients screening for HBV?		
Yes	463	92.0
No	11	2.2
Not sure	12	2.4
Refused to answer	17	3.4
Missing	3	
Do you recommend screening and vaccination for sexual partners of persons with HBV infection?		
Yes	436	86.4
No	12	2.4
Not sure	28	5.6
Refused to answer	28	5.6
Missing	2	
Do you encourage the family members and other close personal contacts of persons with HBV infection persons to be tested and vaccinated?		
Yes	424	84.3
No	17	3.4
Not sure	42	8.3
Refused to answer	20	4.0
Missing	3	
Do you refer patients with HBV infection to a specialist (infectionist, hepatologist, gastroenterologist)?		
Yes	460	91.6
No	8	1.6
Not sure	14	2.8
Refused to answer	20	4.0
Missing	4	
“Do you recommend patients with HBV infection to take hepatoprotective medications?”		
Yes	205	40.7
No	136	27.0
Not sure	123	24.4
Refused to answer	40	7.9
Missing	2	

Abbreviation: HBV, hepatitis B virus.

**TABLE 4 ∣:** Factors associated with total overall HBV knowledge score (low, medium, high) based on bivariate ordinal logistic regression, Georgia 2022.

Characteristic	Total	Knowledge level						OR (medium/high)	95% CI
		Low (ref)		Medium		High			
		*n*	% (row)	*N*	% (row)	*n*	% (row)		
Sex									
Male	35	16	45.71	10	28.57	9	25.71	1 (ref)	
Female	469	171	36.46	150	31.98	148	31.56	1.28	0.81–2.03
Age									
< 30	10	4	40	4	40	2	20	1	
30–39	46	13	28.26	15	32.61	18	39.13	0.81	0.38–1.76
40–49	127	38	29.92	44	34.65	45	35.43	0.46	0.23–0.88
50–59	153	44	28.76	54	35.29	55	35.95	0.96	0.57–1.65
≥ 60	170	89	52.35	43	25.29	38	22.35	0.79	0.53–1.16

Abbreviations: 95% CI, 95% confidence interval; HBV, hepatitis B virus; OR, odds ratio; ref, reference category.

**TABLE 5 ∣:** R Association between overall hepatitis B knowledge score^a^ (low, medium, high) and hepatitis B-related medical practices, Georgia 2022.

	Total	Yes		No (ref)		aOR^b^	95% CI
		*n*	% (row)	*n*	% (row)		
Do you recommend a caesarean section for HBV-positive pregnant women?							
Level of knowledge							
Low	126	94	74.60	32	25.40	1	
Middle	118	76	64.41	42	35.59	0.61	0.35–1.07
High	115	68	59.13	47	40.87	0.47	0.27–0.82
Do you recommend patients with HBV infection to take hepatoprotective medications?							
Level of knowledge							
Low	129	93	72.09	36	27.91	1	
Middle	104	58	55.77	46	44.23	0.55	0.31–0.96
High	108	54	50.00	54	50.00	0.43	0.25–0.73
Have you been vaccinated against HBV?							
Level of knowledge							
Low	165	39	23.64	126	76.36	1	
Middle	146	51	34.93	95	65.07	1.55	0.92–2.61
High	135	42	31.11	93	68.89	1.25	0.73–2.14
Do you recommend HBV-positive mothers to avoid breastfeeding?							
Level of knowledge							
Low	129	53	41.09	76	58.91	1	
Middle	126	48	38.10	78	61.90	0.88	0.53–1.47
High	136	39	28.68	97	71.32	0.57	0.34–0.96
Do you recommend screening and vaccination for sexual partners of HBV-infected persons?^c^							
Level of knowledge							
Low	159	152	95.60	7	4.40	—	—
Middle	153	150	98.04	3	1.96	—	—
High	136	134	98.53	2	1.47	—	—
Do you recommend to your patients to receive screening for HBV?^c^							
Level of knowledge							
Low	178	169	94.94	9	5.06	—	—
Middle	152	151	99.35	1	0.66	—	—
High	144	143	99.31	1	0.69	—	—

Abbreviations: 95% CI, 95% confidence interval; aOR, adjusted odds ratio; HBV, hepatitis B virus; ref, reference category.

a The knowledge level of each participant was assessed by calculating individual knowledge scores by counting each correct answer as one point; total knowledge scores ranging from 0 to 28 were calculated (based on the number of correct answers in the ‘knowledge’ section of the survey). The knowledge scores of the participants were then classified as low (0–16), middle (17–19) and high (20–28) based on the tertile distribution.

b Adjusted for age and sex.

c Due to the low frequencies in the ‘NO’ category we did not proceed with the regression model.

## Data Availability

The data that support the findings of this study are available on request from the corresponding author. The data are not publicly available due to privacy or ethical restrictions.
