# Supplementary material for: Evaluation of Knowledge, Attitudes and Practices for Hepatitis B Virus Infection Among Primary Healthcare Physicians in Georgia
Source: J Viral Hepat. Author manuscript; Available in PMC 2025 Dec 1. (PMC11578783; doi:10.1111/jvh.14011)
Supplement: Supplementary table [file NIHMS2028798-supplement-Supplementary_table.docx]

# Appendix 2. Supplementary Tables

Supplementary Table 1. Primary Healthcare Physicians’ Attitudes and Perception of Hepatitis B, Georgia, 2022

| **Questions** | **Primary health care physicians (N-506)** | |
| --- | --- | --- |
|  | **n** | **%** |
| **HBV is a serious public health problem in Georgia** | | |
| Strongly agree | 230 | 45.6 |
| Agree | 186 | 36.9 |
| Neutral | 36 | 7.1 |
| Disagree | 9 | 1.8 |
| Strongly disagree | 19 | 3.8 |
| I do not know | 24 | 4.8 |
| Missing | 2 |  |
| **I am confident in conducting diagnostic tests for patients with HBV infection** | | |
| Strongly agree | 144 | 28.5 |
| Agree | 223 | 44.2 |
| Neutral | 41 | 8.1 |
| Disagree | 23 | 4.6 |
| Strongly disagree | 11 | 2.2 |
| I do not know | 63 | 12.4 |
| Missing | 1 |  |
| **I am confident in managing patients with HBV infection** | | |
| Strongly agree | 59 | 11.7 |
| Agree | 129 | 25.6 |
| Neutral | 83 | 16.5 |
| Disagree | 117 | 23.2 |
| Strongly disagree | 31 | 6.2 |
| I do not know | 85 | 16.8 |
| Missing | 2 |  |
| **I want to get trainings to learn more about the management of patients with HBV infection** | | |
| Strongly agree | 273 | 54.1 |
| Agree | 197 | 39.0 |
| Neutral | 21 | 4.1 |
| Disagree | 4 | 0.8 |
| Strongly disagree | 5 | 1.0 |
| I do not know | 5 | 1.0 |
| Missing | 1 |  |
| **I think that treatment for chronic HBV infection is very expensive** | | |
| Strongly agree | 106 | 20.9 |
| Agree | 191 | 37.7 |
| Neutral | 48 | 9.5 |
| Disagree | 21 | 4.2 |
| Strongly disagree | 8 | 1.6 |
| I do not know | 132 | 26.1 |
| **I think that treatment for chronic HBV infection has many side effects** | | |
| Strongly agree | 34 | 6.7 |
| Agree | 133 | 26.3 |
| Neutral | 115 | 22.7 |
| Disagree | 54 | 10.7 |
| Strongly disagree | 29 | 5.7 |
| I do not know | 141 | 27.9 |
| **I am concerned about getting HBV from my patients** | | |
| Strongly agree | 26 | 5.1 |
| Agree | 52 | 10.3 |
| Neutral | 73 | 14.4 |
| Disagree | 294 | 58.1 |
| Strongly disagree | 47 | 9.3 |
| I do not know | 14 | 2.8 |
| **The HBV vaccine is safe** | | |
| Strongly agree | 192 | 37.9 |
| Agree | 230 | 45.5 |
| Neutral | 30 | 5.9 |
| Disagree | 12 | 2.4 |
| Strongly disagree | 9 | 1.8 |
| I do not know | 33 | 6.5 |

Abbreviations: HBV, hepatitis B virus
