# Supplementary material for: Evaluation of Knowledge, Attitudes and Practices for Hepatitis B Virus Infection Among Primary Healthcare Physicians in Georgia
Source: J Viral Hepat. Author manuscript; Available in PMC 2025 Dec 1. (PMC11578783; doi:10.1111/jvh.14011)
Supplement: Appendix [file NIHMS2028798-supplement-Appendix.docx]

# Appendix 1- Study Questionnaire

**Evaluation of knowledge, attitudes, and practices for hepatitis B virus infection among primary healthcare physicians- Questionnaire**

General information

1) What is your age range (in years)?

a) <30

b) 30-39

c) 40-49

d) 50-59

e) ≥60

2) What is your sex?

a) Male

b) Female

3) How long have you been a physician?

a) ≤5 years

b) 6-10 years

c) 11-15 years

d) ≥15 years

e) Refused to answer

4) Except for the certification in “Family Medicine” do you have other specialty trainings?

a) Infectious Disease

b) Gastroenterology

c) Internal Medicine

d) Other ____________________________

5) What is your salary range as a physician (GEL per month)?

a) <400

b) 400-800

c) 801-1200

d) >1200

e) Refuse to answer

6) How many patients do you see with hepatitis B virus (HBV) infection per year?

a) 0-10

b) 10-50

c) 51-100

d) >100

Part I - Knowledge

1) Which of the below can cause hepatitis (inflammation of the liver)?

a) Virus

b) Alcohol

c) Drugs

**d) All above mentioned**

e) None of the above

f) Other ____________

g) Don’t know

h) Refuse to answer

2) Infection with HBV can cause:

a) Liver cirrhosis and cancer

b) Arthritis

c) Vasculitis

d) Glomerulonephritis and renal failure

e) **All above mentioned**

f) None above mentioned

g) Don’t know

h) Refused to answer

3) What percentage of adult (more than 17 years old) Georgian population has chronic HBV infection?

a) <1%

**b) 1-5%**

c) 5-10%

d) >10%

e) Don’t know

f) Refused to answer

Risk Factors

4) Which groups of people are considered high-risk for HBV exposure:

a) Healthcare workers

b) People with multiple blood transfusions

c) People who inject drugs

d) Commercial sex-workers

e) Children from HBV positive mothers

f) Persons on hemodialysis

g) **All above mentioned**

h) None above mentioned

i) Don’t know

j) Refuse to answer

How is HBV transmitted? ( “Yes” and “No” options were listed after each possible transmission mode for questions 5-13)

5) Droplets (coughing, sneezing, respiratory secretions)

6) Food

7) **Blood**

8) **Sexual contact**

9) Handshake with an infected person

10) **Sharing household objects like razors or toothbrushes**

11) **Sharing needles or syringes**

12) Touching items in public places (doorknobs, handles in transport, unhygienic toilets)

13) **Mother to child during birth**

14) Choose the correct answer: The risk of HBV transmission by a needle-stick is

a) Lower than HIV

b) Lower than HCV

c) **Higher than HCV**

d) Don’t know

e) Refused to answer

15) Persons with HBV infection can:

a) Donate blood

b) Donate an organ

c) Donate both

d) **Donate none**

e) Don’t know

f) Refused to answer

Diagnosis and Treatment

16) What is the incubation period for HBV infection:

a) 1-3 weeks

b) **1-6 months**

c) 7-12 months

d) Don’t know

e) Refused to answer

17) Acute HBV infection can be presented with:

a) Jaundice

b) Nonspecific symptoms (fever, nausea, fatigue, etc)

c) Fulminant hepatitis (ascites, encephalopathy, coagulopathy etc.)

d) Without symptoms

e) **All above mentioned**

f) Don’t know

g) Refused to answer

18) What proportion of adults who have acute HBV infection spontaneously clear the infection?

a) <5%

b) 20%

c) **90-95%**

d) Don’t know

e) Refused to answer

19) What test is usually used first to confirm current HBV infection?

a) Anti-HBc (Hepatitis B core antibodies)

b) **HBsAg (Hepatitis B surface antigen)**

c) HBeAg (Hepatitis B envelope antigen)

d) Anti-HBs (Hepatitis B surface antibody)

e) PCR HBV-DNA (PCR for identification of viral DNA)

f) Other_______________

g) Don’t know

h) Refused to answer

20) Chronic HBV infection can be cured:

a) Yes

b) **No**

c) Don’t know

d) Refused to answer

21) Do all persons with chronic HBV infection patients without antiviral treatment develop liver cirrhosis or cancer?

a) Yes

b) **No**

c) Don’t know

d) Refused to answer

22) Which diagnostic evaluation is essential to determine the need of antiviral treatment for patients with chronic HBV infection?

a) Liver function tests / liver enzymes

b) HBV viral load (PCR HBV-DNA quantitative test)

c) Liver fibrosis

d) **All above mentioned**

e) All chronic HBV patients need antiviral treatment

f) Don’t know

g) Refuse to answer

23) If a patient is not on treatment, what is the recommended interval (frequency) of surveillance/monitoring:

a) Every 1 to 3 months

b) **Every 6 to 12 months**

c) Every 2-3 years

d) Every 5-10 years

e) Does not need monitoring

f) Don’t know

g) Refuse to answer

Questions related to HBV vaccination

24) How many doses of HBV vaccine are required for complete protection (choose the best answer)?

a) 1 dose

**b) 3 doses**

c) 6 doses

d) Don’t know

e) Refused to answer

25) Since what year has HBV vaccine been included in the routine vaccination schedule in Georgia?

a) Since 1960

b) Since 1993

**c) Since 2001**

d) Since 2016

e) Don’t know

f) Refused to answer

26) Which of the following are contraindications to HBV vaccination?

a) Allergic rhino-sinusitis

b) Neurologic disorders

c) Liver diseases

d) Age >60

e) All above mentioned

**f) None above mentioned**

g) Don’t know

h) Refused to answer

27) How effective is HBV vaccine in preventing infection?

a) <60%

b) 60-80%

**c) >90%**

d) Don’t know

e) Refused to answer

28) What is recommended to prevent mother to child transmission in mothers with chronic HBV?

a) Cesarean section

**b) Hepatitis B immunoglobulin within first 24 hours after birth and timely birth dose vaccination**

c) Withholding breastfeeding

d) All above mentioned

e) None above mentioned

f) Don’t know

g) Refused to answer

Part II - Attitudes and Perceptions

Statements: Strongly agree, Agree, Neutral, Disagree, Strongly disagree, I do not know

1) HBV is serious public health problem in the Georgia

2) I think that all persons living in Georgia should be tested for HBV

3) I am confident in conducting diagnostic tests for patients with HBV infection

4) I am confident in managing patients with HBV infection

5) If I find out that my patient has HBV I refer her/him immediately to the specialists

6) I think that treatment for chronic HBV infection is very expensive

7) I think that treatment for chronic HBV infection has many side effects

8) I want to get trainings to learn more about management of patients with HBV infection

9) If I can be trained, I would feel comfortable managing patients with HBV infection

10) I am confident in counseling patients about the prevention of HBV infection

11) I am concerned about getting HBV from my patients

12) HBV vaccine is safe

13) As a healthcare provider, it is necessary for me to receive HBV vaccine

14) I think stigma exists for patients with HBV infection in Georgia

Part III - Practice related questions

Answers: Yes, no, not sure, refuse to answer

1) Have you been screened for HBV?

2) Have you been vaccinated against HBV?

3) Have you ever participated in a health education program related to HBV?

4) Do you recommend to your patients screening for HBV?

5) Do you recommend screening and vaccination for sexual partners of persons with HBV infection?

6) Do you encourage the family members and other close personal contacts of persons with HBV infection to be tested and vaccinated?

7) Do you counsel your patients to ensure equipment is disinfected or newly opened when they go to the barber, salon, or have piercings?

8) Would you recommend a patient who has just been diagnosed with HBV infection to continue further diagnostic investigations?

9) Do you refer patients with HBV infection to the specialist (Infectionist, Hepatologist, Gastroenterologist)?

10) Do you recommend patients with HBV infection take medications only under physician’s instructions?

11) Do you recommend patients with HBV infection to avoid sharing food/utensils/water with others?

12) Do you recommend patients with HBV infection to take hepatoprotective medications?

13) Do you recommend a strict low-fat diet to patients with HBV infection?

14) Do you recommend restricted physical activity to patients with HBV infection?

15) Do you recommend patients with HBV infection avoid alcohol?

16) Do you recommend persons with chronic HBV infection should be monitored regardless of treatment status?

17) Do you recommend caesarian section for HBV positive pregnant women?

18) Do you recommend HBV positive mothers avoid breastfeeding?

# Appendix 2. Supplemenmtary Tables

Supplementary Table 1. Primary Healthcare Physicians’ Attitudes and Perception of Hepatitis B, Georgia, 2022

| **Questions** | **Primary health care physicians (N-506)** | |
| --- | --- | --- |
|  | **n** | **%** |
| **HBV is a serious public health problem in Georgia** | | |
| Strongly agree | 230 | 45.6 |
| Agree | 186 | 36.9 |
| Neutral | 36 | 7.1 |
| Disagree | 9 | 1.8 |
| Strongly disagree | 19 | 3.8 |
| I do not know | 24 | 4.8 |
| Missing | 2 |  |
| **I am confident in conducting diagnostic tests for patients with HBV infection** | | |
| Strongly agree | 144 | 28.5 |
| Agree | 223 | 44.2 |
| Neutral | 41 | 8.1 |
| Disagree | 23 | 4.6 |
| Strongly disagree | 11 | 2.2 |
| I do not know | 63 | 12.4 |
| Missing | 1 |  |
| **I am confident in managing patients with HBV infection** | | |
| Strongly agree | 59 | 11.7 |
| Agree | 129 | 25.6 |
| Neutral | 83 | 16.5 |
| Disagree | 117 | 23.2 |
| Strongly disagree | 31 | 6.2 |
| I do not know | 85 | 16.8 |
| Missing | 2 |  |
| **I want to get trainings to learn more about the management of patients with HBV infection** | | |
| Strongly agree | 273 | 54.1 |
| Agree | 197 | 39.0 |
| Neutral | 21 | 4.1 |
| Disagree | 4 | 0.8 |
| Strongly disagree | 5 | 1.0 |
| I do not know | 5 | 1.0 |
| Missing | 1 |  |
| **I think that treatment for chronic HBV infection is very expensive** | | |
| Strongly agree | 106 | 20.9 |
| Agree | 191 | 37.7 |
| Neutral | 48 | 9.5 |
| Disagree | 21 | 4.2 |
| Strongly disagree | 8 | 1.6 |
| I do not know | 132 | 26.1 |
| **I think that treatment for chronic HBV infection has many side effects** | | |
| Strongly agree | 34 | 6.7 |
| Agree | 133 | 26.3 |
| Neutral | 115 | 22.7 |
| Disagree | 54 | 10.7 |
| Strongly disagree | 29 | 5.7 |
| I do not know | 141 | 27.9 |
| **I am concerned about getting HBV from my patients** | | |
| Strongly agree | 26 | 5.1 |
| Agree | 52 | 10.3 |
| Neutral | 73 | 14.4 |
| Disagree | 294 | 58.1 |
| Strongly disagree | 47 | 9.3 |
| I do not know | 14 | 2.8 |
| **The HBV vaccine is safe** | | |
| Strongly agree | 192 | 37.9 |
| Agree | 230 | 45.5 |
| Neutral | 30 | 5.9 |
| Disagree | 12 | 2.4 |
| Strongly disagree | 9 | 1.8 |
| I do not know | 33 | 6.5 |

Abbreviations: HBV, hepatitis B virus
